# Association between physical activity and health-related quality of life in children: a cross-sectional study

**DOI:** 10.1186/s12955-016-0474-y

**Published:** 2016-05-04

**Authors:** Sharifah Wajihah Wafa bte Syed Saadun Tarek Wafa, Mohd Razif bin Shahril, Aryati bte Ahmad, Laila Ruwaida bte Zainuddin, Karimah Fakhriah bte Ismail, Myat Moe Thwe Aung, Noor Aini bte Mohd Yusoff

**Affiliations:** Faculty of Health Sciences, Universiti Sultan Zainal Abidin (UniSZA), Gong Badak Campus, 21300 Kuala Terengganu, Terengganu Darul Iman Malaysia; Faculty of Medicine, Universiti Sultan Zainal Abidin (UniSZA), Medical Campus, Jalan Sultan Mahmud, 20400 Kuala Terengganu, Terengganu Darul Iman Malaysia

**Keywords:** Childhood, Obesity, Physical activity, Quality of life, Accelerometer

## Abstract

**Background:**

Research suggests that physical activity plays a role to improve health related- quality of life (QoL), however studies examining the association between physical activity and HRQOL are limited in the paediatric literature. The aim of this study is to explore the relationship between physical activity and HRQoL among Malaysian children.

**Methods:**

Participants (*n* = 78 normal weight; 78 obese children) aged 9–11 years completed a validated quality of life (QoL) inventory and wore an accelerometer to objectively measure physical activity for 1 week.

**Results:**

Psychosocial Health domain and Total QoL (all *p* < 0.05) were significantly lower for obese compared to normal weight children. Children who spent more time in sedentary behaviour had significantly lower QoL on Psychosocial Health domain and Total QoL except for the Physical Health domain. There was also a strong positive correlation between QoL and moderate-vigorous physical activity (MVPA) indicating that children who are physically active have a better quality of life.

**Conclusions:**

Physical activity promotion should be emphasised to improve QoL in children.

## Background

Over the past decades, numerous well-established studies have reported the benefits of physical activity (PA) that include skeletal health, obesity prevention and psychological health in children [1–4]. Recent WHO recommendations on physical activity for health aimed at children and adolescents age 5–17 years stated that they should participate in at least 60 min of moderate-to-vigorous physical activity (MVPA) 5 days a week [4]. Despite the potential benefits, as an impact of lifestyle on today’s societies, this PA guideline has not been achieved leading to excess weight gain among the children [5]. A recent study by Naidu et al. (2013) examined the prevalence of overweight and obesity in 7,749 representative samples of Malaysian children between 7 and 12 years old [6]. The study reported that the prevalence of overweight and obesity children in Malaysia was 19.9 % and it was concluded that one out of five of 7–12 year-old-children in Malaysia were overweight or obese [6].

Most studies of habitual physical activity in children suggest that the overweight and obese children are less active [7–10]. A study reported that Malaysian children have exceptionally low MVPA levels and very high levels of sedentary behaviour [11]. Lee et al. (2014) reported that Malaysian children spend half of the time on screen-related activities that includes watching television, playing video games, and using the computer [12]. Besides that, a positive association between excess weight and decrease in health-related quality of life (HRQoL) in children has also been documented [13]. There is an emerging body of evidence that obesity in general has been linked to low self-image, low self-confidence and even depression in some obese children which may impact quality of life [14]. Although the obese children reported a lower QoL [8, 13–21], few studies are available to examine whether low physical activity of obese children is associated with decreased HRQoL among them. There is a study that supports children who participate regularly in physical activity are more likely to have better HRQoL than children who never participate in physical activity [22].

Previous study showed that intervention for the treatment of childhood obesity had a greater impact on QoL [8]. However, the study did not report physical activity might play a role in this regard. Furthermore, the association between PA and QoL has not been studied among children living in low- and low-middle-income countries such as Malaysia, which have the highest overweight/obesity prevalence in Asian countries. Therefore, the main aim of this study was to explore the association between objectively measured physical activity and HRQoL in Malaysian school children aged 9 to 11 years old.

## Methods

### Participants

This study was a cross-sectional study conducted at five primary schools in Kuala Terengganu which were selected via convenience sampling design. Sample size for the present study was based on a previous study which found a statistically significant difference in total physical activity between obese and non-obese children of the same age [23]. We calculated that a minimum of 40 pairs, with 20 pairs of boys and 20 pairs of girls were required to have a >90 % power at the 5 % significance level to detect a mean difference of 100 accelerometry counts/min/day (a measure of total volume of physical activity, and this difference was roughly equivalent to the difference between boys and girls in most previous studies) [24] for each sex.

From these five schools, screening of weight status to determine eligibility was carried out in a total 500 school children (305 boys, 195 girls) aged 9–11 years old. School children were categorised into underweight, normal weight, overweight and obese group relative to WHO 2007 reference chart. Obesity was defined as BMI z-score > +2SD, overweight as BMI z-score > +1SD, normal weight was defined as BMI z-score > −2SD and < +1SD and underweight as BMI z-score < −2SD on age- and gender- specific. Study participants were included if they were either obese or normal weight as defined above. Underweight and overweight children were excluded from the study sample. Other exclusions were children with serious chronic or acute illness which might affect their QoL. The total number of school children who did not fulfil the inclusion criteria and/or did not consent was 250 leaving 250 (144 boys, 106 girls) eligible consenting participant. A pre-planned paired analysis of HRQoL between obese and normal weight children, with pair matching for same age and gender yielded 78 pairs with 43 paired comparisons in boys and 35 paired comparison in girls.

The study was approved by the Universiti Sultan Zainal Abidin (UniSZA) Human Research Ethics Committee [UniSZA.N/1/628-1 (28)], and written informed consent was obtained from parents and assent from the children. Also, approval to conduct the study in government public schools was obtained from the Ministry of Education Malaysia.

### Anthropometric measurements

Participants’ height was measured using portable stadiometer to the nearest 0.1 cm. Weight was measured to the nearest 0.1 kg using the digital weight scale (Seca Robusta 813) with children in light indoor clothing. Using height, weight, age, and sex data, BMI z-score were calculated for each individual according to the WHO (2007) reference and participants were categorized based on weight status classification (i.e., normal weight, or obese). For purposes of the current study, our normal weight group was composed of participants whose BMI z-score ranked ≥ -2SD and < +1SD, whereas the obese group had a BMI z-score > + 2SD.

### Measurement of physical activity and sedentary behaviour

Habitual physical activity and sedentary behaviour were measured objectively during the waking hours using CSA/MTI GT1M accelerometer (The Actigraph, Fort Walton Beach, Florida, USA) for 5 days. Accelerometry data were included so long as at least 5 days of monitoring with at least 10 h per day were obtained. The accelerometers were set to record activity in 15 s epochs, collapsed to 1 min when cut-points were applied to measure the intensity of physical activity and sedentary behaviour. Accelerometry data were summarised using cut-points to define time spent in sedentary behaviour (<1100 cpm) [25] light intensity physical activity (1100–3200 cpm), and moderate to vigorous intensity physical activity (MVPA) (>3200 cpm) [26]. These are all empirically determined cut-off points based on previous paediatric validation and calibrated studies [25, 26].

Participants were instructed to wear the accelerometer around the waist on an adjustable elastic belt and worn over the right hip under clothing. They also recorded the time the monitor was attached in a diary and removed each day including at other times that the monitor was removed during the day, for example when bathing. Data were downloaded and handled manually.

### Health-related quality of life

Health-related QoL was measured using the Malay version of Health-related quality of life Inventory™ Version 4.0 (PedsQL) [21]. Participating children completed the PedsQL at school in the presence of a researcher. In brief, the PedsQL is a child self-report consisting of 23 items made up of physical (eight items), social (five items), emotional (five items) and school functioning (five items) components [27]. This measure was scored using a five-point scale (0 = never; 1 = almost never; 2 = sometimes; 3 = often; 4 = always). These items were then reverse scored on a scale of 1–100 (i.e., 0 = 100, 1 = 75, 2 = 50, 3 = 25, and 4 = 0), so that higher scores indicate better QoL. Total scores from all 23 items were calculated to provide an overall measure of the QoL, and two domains were calculated, for physical health (from the sum of the physical components) and psychosocial health (from the sum of the social, emotional, and school functioning components.

### Statistical analysis

All statistical analyses were conducted using statistical package SPSS version 20.0. Categorical variables are presented as frequency (percentage) and continuous variables are presented as means and standard deviation (SD). Paired *t*-test was used to compare mean QoL scores, daily minutes MVPA and sedentary behaviour between obese and normal weight children. Multiple linear regression was applied to determine the associated factors of health-related quality of life. Variables chosen for multiple linear regression analysis using stepwise method were decided not only based on statistical significance in univariable analysis (p <0.25) but also on principles of parsimony and biological plausibility. Final results were presented with crude and adjusted regression coefficients with 95 % confidence interval (CI) and corresponding *p*-values. A *p*-value of less than 0.05 was regarded as statistically significant.

## Results

### Sample characteristics

The sample consisted of 78 obese children pair matched with 78 normal weight children, with the mean age of the study sample being 9.9 (SD 0.5) years. The anthropometrics characteristics, daily minutes MVPA and sedentary behaviour and QoL scores are presented in Table 1. The obese sample had an average BMI z-score relative to WHO, 2007 reference data falling at 2.9 (SD 0.6) kg/m^2^ while average BMI z-score for normal weight children was − 0.2 (SD 0.7).

**Table 1:** Characteristics of participants

Variables	Normal weight children (*n* = 78)	Obese children (*n* = 78)	*P*- value*	Full sample (*n* = 156)
Male/Female	43/35	43/35		86/70
Age (years)	10.0 (0.6)	9.7 (0.5)	**<0.001**	9.9 (0.5)
Anthropometric measurements				
Weight (kg)	30.0 (4.4)	53.7 (13.0)	**<0.001**	41.9 (15.3)
Height (cm)	134.7 (6.3)	139.4 (10.1)	**<0.001**	137.0 (8.7)
BMI	16.4 (1.5)	27.3 (4.2)	**<0.001**	21.9 (6.3)
BMI z-score	−0.2 (0.7)	2.9 (0.6)	**<0.001**	1.4 (1.7)
Habitual Physical Activity * % of monitored time during the day*	384.7 (129.7)	329.4 (130.0)	**0.01**	357.0 (132.4)
Total physical activity (cpm)				
Sedentary behaviour	87.2 (4.8)	90.9 (4.6)	**<0.001**	89.1 (5.0)
Light-intensity physical activity	10.3 (4.1)	8.4 (4.2)	**0.01**	9.3(4.2)
MVPA	1.4 (1.1)	0.7 (0.7)	**<0.001**	1.0 (1.0)
Quality of life				
PedsQL Physical Health	62.3 (22.6)	68. 2 (19.3)	0.08	65.3 (21.2)
PedsQL Psychosocial Health	77.9 (16.1)	65.3 (14.6)	**<0.001**	71.6 (16.6)
Emotional functioning	76.7 (19.3)	64.0 (22.0)	**<0.001**	70.3 (21.6)
Social functioning	80.2 (21.5)	66.4 (20.5)	**<0.001**	73.3 (22.0)
School functioning	77.2 (16.5)	65.4 (19.3)	**<0.001**	71.3 (18.9)
PedsQL Total score	74.7 (11.8)	65.9 (14.2)	**<0.001**	70.3 (13.8)

*Paired *t*-test. Significant *p*-values (<0.05) in bold

For physical activity levels, the accelerometer was worn over 5 days on average for a mean of 14.6 waking hours per day. The results showed that the proportion of monitored time spent in sedentary behaviour for this sample was high at around 89 % of the waking day, or about 13 waking hours of the day. Participation in moderate to vigorous physical activity was extremely low at an average of 1 % of monitored time during the day, equivalent to about 9 min per day, on average. In addition, mean PedsQL Total Score of the sample was 70.3 (SD 13.8). The average score Physical Health domain and Psychosocial Health domain were 65.3 (SD 21.2) and 71.5 (SD 13.8), respectively.

### Physical activity by weight status

In the evaluation between physical activity and weight status, there appeared to be statistically significant differences between the normal and obese children. Normal weight children reported significantly higher participation in MVPA and lower participation in sedentary behaviours than obese children as shown in Table 1.

### Mean scores of QoL domains by weight status

There were statistically significant differences between the normal and obese children for the Psychosocial Health domain and PedsQL Total Score as shown in Table 1. Normal weight children reported significantly better scores than obese children. However, there was no significant difference between the groups for the Physical Health domain.

### Formal linear regression association between physical activity levels and QoL

Children who spend more time in sedentary behaviour or less time in MVPA were associated with lower Psychosocial Health domain and PedsQL Total Score in unadjusted model but not the adjusted model (Table 2). Lower BMI was related to better Psychosocial Health domain (adjusted: Stand. coeff. − 0.221) in both non-adjusted and adjusted analysis. However, the same association between BMI and PedsQL Total Score was limited only in unadjusted analysis. Interestingly, time spent in sedentary behaviour and MVPA were not predictors for better Physical Health domain. Gender of children, on the other hand, had no association with any of the QoL outcomes.

**Table 2:** The associated factors of health-related quality of life (*n* =156)

	Psychosocial health *n* = 156 Reg. coeff. (95 % CI)	*P*	Physical health *n* = 156 Reg. coeff. (95 % CI)	*P*	PedsQL total score *n* = 156 Reg. coeff. (95 % CI)	*P*
BMI (kg/m^2^)						
^a^Unadjusted	−0.83 (−1.23,−0.43)	**<0.001**	0.41 (−0.13, 0.94)	0.134	−0.57 (−0.91,−0.23)	**0.001**
^b^Adjusted	−0.58 (−1.03,−0.13)	**0.012**	0.10 (−0.27, 0.97)b	0.265	−0.31 (−0.69, 0.07)	0.109
Physical activity^c^ (cpm)						
^a^Unadjusted	0.03 (0.02, 0.05)	**0.001**	−0.02 (−0.04, 0.01)	0.140	0.02 (0.01, 0.04)	**0.006**
^b^Adjusted	−0.01 (−0.06, 0.05)	0.916	−0.02 (−0.09, 0.06)b	0.617	−0.02 (−0.07, 0.02)	0.316
Sedentary behaviour (min)						
^a^Unadjusted	−6.78(−10.10, −3.46)	**<0.001**	3.70 (−0.73, 8.12)	0.101	−5.01 (−7.79,−2.21)	**0.001**
^b^Adjusted	−3.35 (−13.19, 6.47)	0.501	1.48 (−11.98, 14.95)b	0.828	−5.82 (−14.15, 2.49)	0.169
MVPA (min)						
^a^Unadjusted	0.55 (0.27, 0.83)	**<0.001**	−0.15 (−0.53, 0.23)	0.431	0.41 (0.18, 0.65)	**0.001**
^b^Adjusted	0.24 (−0.18, 0.66)	0.262	0.24 (−0.34, 0.81)b	0.419	0.25 (−0.11, 0.60)	0.175
Adjusted R^2^	0.126		−0.004		0.094	

^a^Crude regression coefficient by simple linear regression, ^b^Adjusted regression coefficient by multiple linear regression

The models reasonably fitted well. Model assumptions were met. There were no interaction and multicollinearity problems

Significant *p*-values (<0.05) in bold; ^c^ Accelerometer measured (count per minute)

## Discussion

Participants in this study spent about 13 h per day in sedentary activity. In contrast, participation in MVPA was extremely low with a mean time spent about 9 min per day. Meanwhile the QoL Total Scores of obese children was significantly lower than the normal weight children and it was below the values reported for thalassaemia patients in Malaysia [28]. Furthermore, the results also showed that the mean score of QoL was at the lower end of the range described previously for children with other chronic and disabling conditions from western societies [14, 27].

The present study showed a negative relationship between QoL and BMI. Children who are obese had lower QoL on Psychosocial Health domain and Total Score except for the Physical Health domain. This is in line with previous studies reported that obese children have lowered health-related quality of life compared to their normal weight peers [16, 20, 21, 29, 30]. A recent systematic review has shown that being obese can have a significant adverse effect on a child’s quality of life [13]. It has been shown that the greater the severity of obesity, the poorer quality of life that child will experience.

Apart from BMI that showed a relationship to QoL, children who were spent more time in sedentary behaviour had significantly lower QoL on Psychosocial Health domain and Total Score except for the Physical Health domain. We also found a positive relationship between QoL and MVPA indicating that children who are physically active have a better quality of life. However, gender and BMI affects this relationship between QoL and physical activity. After the adjusted analyses, there appear to be no relationship between QoL and physical activity. Interestingly, being a male or female and normal weight or obese seems important for this relationship to occur as there was a lack of adjusted linear relationship between physical activity and QoL. These findings lend further support on the existing evidence base of the relationships among gender, BMI, and QoL [31]. In contrast, Shoup’s study using the same measure of QoL and physical activity in a younger (8–12 years) group of children reported that less physically active children, irrespective of weight status had significantly lower psychosocial and total QoL [22]. This discrepancy may be explained by cultural differences that might influence differences in relationship between quality of life and physical activity.

There are a number of strengths and limitations of this study. The main strengths of the present study were homogeneous sample of the community sample studied and the pair-matched design which allowed key variables (age and gender) to be controlled. Apart from that, the study used a validated multidimensional assessment of QoL for children and objectively measured physical activity. In addition, the study is the first to examine the association between physical activity and QoL in children from low-to-middle-income countries. The primary limitation of the study is a cross-sectional design that does not allow determining the causality of the relationship between QoL and physical activity. Hence, the data presented in this study represents merely a snapshot of information on physical activity and QoL in children.

## Conclusion

In summary, this study provides evidence that physical activity might have a positive relationship with psychosocial and total QoL in children but they are dependent on gender and BMI. We conclude that healthy lifestyle interventions for school children should focus on obese children and pay more attention to independent benefits of physical activity that can be used for improving QoL. These findings should be further tested in a large sample and various settings to determine the possible effect of physical activity on QoL in children.

## References

[1] Chimen M, Kennedy A, Nirantharakumar K, Pang TT, Andrews R, Narendran P (2012). What are the health benefits of physical activity in type 1 diabetes mellitus? A literature review. Diabetologia.

[2] Metcalf B, Henley W, Wilkin T (2012). Effectiveness of intervention on physical activity of children: systematic review and meta-analysis of controlled trials with objectively measured outcomes (EarlyBird 54). BMJ.

[3] Müller-Riemenschneider F, Reinhold T, Nocon M, Willich SN (2008). Long-term effectiveness of interventions promoting physical activity: a systematic review. Prev Med (Baltim).

[4] WHO. Global recommendations on physical activity for health. Geneva World Heal Organ. 2010:60. doi:10.1080/11026480410034349 26180873

[5] Jiménez-Pavón D, Kelly JRJ (2010). Associations between objectively measured habitual physical activity and adiposity in children and adolescents: systematic review. Int J Pediatr Obes.

[6] Naidu BM, Mahmud SZ, Ambak R (2013). Overweight among primary school-age children in Malaysia. Asia Pac J Clin Nutr.

[7] McManus AM, Mellecker RR (2012). Physical activity and obese children. J Sport Heal Sci.

[8] Wafa SW, Talib RA, Hamzaid NH (2011). Randomized controlled trial of a good practice approach to treatment of childhood obesity in Malaysia: Malaysian childhood obesity treatment trial (MASCOT). Int J Pediatr Obes.

[9] Hills AP, Andersen LB, Byrne NM (2011). Physical activity and obesity in children. Br J Sports Med.

[10] Carlson JA, Crespo NC, Sallis JF, Patterson RE, Elder JP (2012). Dietary-related and physical activity-related predictors of obesity in children: a 2-year prospective study. Child Obes.

[11] Wafa SW, Hamzaid H, Talib RA, Reilly JJ (2014). Objectively measured habitual physical activity and sedentary behaviour in obese and non-obese Malaysian children. J Trop Pediatr.

[12] Lee S, Wong J, Shanita S, Ismail M, Deurenberg P, Poh B (2014). Daily physical activity and screen time, but Not other sedentary activities, Are associated with measures of obesity during childhood. Int J Environ Res Public Health.

[13] Buttitta M, Iliescu C, Rousseau A, Guerrien A (2014). Quality of life in overweight and obese children and adolescents: a literature review. Qual Life Res.

[14] Griffiths LJ, Parsons TJ, Hill AJ (2010). Self-esteem and quality of life in obese children and adolescents: a systematic review. Int J Pediatr Obes.

[15] Abdel-Aziz EA, Hamza RT, Youssef AM, Mohammed FM (2014). Health related quality of life and psychological problems in Egyptian children with simple obesity in relation to body mass index. Egypt J Med Hum Genet.

[16] Riazi A, Shakoor S, Dundas I, Eiser C, McKenzie SA (2010). Health-related quality of life in a clinical sample of obese children and adolescents. Health Qual Life Outcomes.

[17] Finne E, Reinehr T, Schaefer A, Winkel K, Kolip P (2013). Health-related quality of life in overweight German children and adolescents: do treatment-seeking youth have lower quality of life levels? comparison of a clinical sample with the general population using a multilevel model approach. BMC Public Health.

[18] Tsiros MD, Olds T, Buckley JD (2009). Health-related quality of life in obese children and adolescents. Int J Obes (Lond).

[19] Kim HS, Park J, Ma Y, Ham OK (2013). Factors influencing health-related quality of life of overweight and obese children in South Korea. J Sch Nurs.

[20] Williams J, Wake M, Hesketh K, Maher E, Waters E (2005). Health-related quality of life of overweight and obese children. JAMA.

[21] Hamzaid H, Talib RA, Azizi NH, Maamor N, Reilly JJ, Wafa SW (2011). Quality of life of obese children in Malaysia. Int J Pediatr Obes.

[22] Shoup JA, Gattshall M, Dandamudi P, Estabrooks P (2008). Physical activity, quality of life, and weight status in overweight children. Qual Life Res.

[23] Hughes AR, Henderson A, Ortiz-Rodriguez V, Artinou ML, Reilly JJ (2006). Habitual physical activity and sedentary behaviour in a clinical sample of obese children. Int J Obes (Lond).

[24] Riddoch CJ, Mattocks C, Deere K (2007). Objective measurement of levels and patterns of physical activity. Arch Dis Child.

[25] Jackson DM, Reilly JJ, Kelly LA, Montgomery C, Grant S, Paton JY (2003). Objectively measured physical activity in a representative sample of 3- to 4-year-old children. Obes Res.

[26] Puyau MR, Adolph AL, Vohra FA, Butte NF (2002). Validation and calibration of physical activity monitors in children. Obes Res.

[27] Varni JW, Seid M, Kurtin PS (2001). PedsQL 4.0: reliability and validity of the pediatric quality of life inventory version 4.0 generic core scales in healthy and patient populations. Med Care.

[28] Ismail A, Campbell MJ, Ibrahim HM, Jones GL (2006). Health related quality of life in Malaysian children with thalassaemia. Health Qual Life Outcomes.

[29] Schwimmer JB, Burwinkle TM, Varni JW (2003). Health-related quality of life of severely obese children and adolescents. JAMA.

[30] Hughes AR, Farewell K, Harris D, Reilly JJ (2007). Quality of life in a clinical sample of obese children. Int J Obes (Lond).

[31] Choo J, Jeon S, Lee J (2014). Gender differences in health-related quality of life associated with abdominal obesity in a Korean population. BMJ Open.

